# Lamellar α‑Titanium Phosphate Modified with Magnesium Sulfate: Effects on Structural, Thermal, Morphological, and Electrochemical Impedance

**DOI:** 10.1021/acsomega.6c03822

**Published:** 2026-07-14

**Authors:** Gerson Alberto Valencia Albitres, Carlos Magno Fialho Soares, Enzo Erbisti Garcia, Daniela de França da Silva Freitas, Paulo Henrique Picciani, Sibele Piedade Cestari, Luis Claudio Mendes

**Affiliations:** † Instituto de Macromoléculas Professora Eloisa Mano, Centro de Tecnologia, Bloco J, 28125 Universidade Federal do Rio de Janeiro, Ilha do Fundão, Rio de Janeiro 21941-598, Brazil; ‡ Universidade do Minho, Campus de Azurém, Centre for Innovation in Polymer Engineering (PIEP), Guimarães, Braga 4800-058, Portugal

## Abstract

Broadly speaking, hierarchically structured materials are those in which their structural regularity in different length scales can permit the enhancement of their multifunctional properties. α-Titanium phosphate (α-TiP) is considered like a material with a hierarchical arrangement. In this context, this study intended to investigate the effect of the incorporation of magnesium sulfate (MgSO_4_) on α-TiP physicochemical properties. α-TiP was synthesized using titanium isopropoxide and phosphoric acid. Either synthesized or intercalated with ethylamine aqueous solution, MgSO_4_ was added into ETiP via solvent-based and dry handling mixing routes. Structural, thermal, morphological, and electrochemical properties were analyzed. X-ray diffraction revealed that the presence of an ethylamine aqueous solution influenced the interlamellar layer of phosphate. The route of MgSO_4_ intercalation induced variation on its amount inside the phosphate galleries as seen by SEM/EDS. A possible interaction between magnesium salt and ethylamine through hydrogen bond formation was hypothesized. Raman spectroscopy indicated spectral changes depending on the route of magnesium salt incorporation. Spin-lattice (T_1_H) and spin-spin (T_2_H) time relaxations revealed that the role of ethylamine aqueous solution as an intercalator was crucial on the phosphate molecular mobility after addition of MgSO_4_. Electrochemical impedance showed that the ETiP/EMgSO_4_ sample showed the best Nyquist and Bode diagrams. Plot of current versus voltage indicated that solvent-based sample ETiP/EMgSO_4_ showed electrical conductivity while that named dry handling mixing αTiP/MgSO_4_(MF) is an insulator. The ETiP/EMgSO_4_ sample exhibited lower impedance around 2 to 3 orders of magnitude lower than precursors. Ethylamine aqueous solution played a key role in modulating phosphate properties and MgSO_4_ mobility within the phosphate matrix.

## Graphical abstract

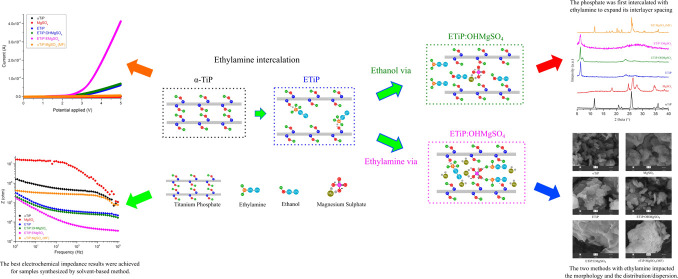

## Introduction

The search for materials with unique characteristics has received considerable attention. Promising hierarchically structured materials—with multiple levels of structural regularity that enhance functionality—have been explored for use as sorbents, electrocatalysts, capacitor electrodes, photoanodes, and sensors.^1,2^ Layered transition metals belong to a class of materials with 2D lamellar structures that offer many applications. Layered tetravalent metal phosphates—such as zirconium and titanium—have received special attention due to their 2D lamellar structure and high surface area, making them attractive for applications in ion exchange, catalysis, ionic conductivity, and ion storage electrodes.^3–5^ Additionally, their interlamellar layers allow functionalization with organic and inorganic species, making them suitable for use as fillers in polymeric nanocomposites.^6–9^ Han et al.^10^ developed a composite ceramic membrane based on lithium/aluminum/titanium phosphate (LATP) for selective lithium recovery. The authors reported that after a three-stage electrodialysis process, the membrane was capable of reducing the magnesium-to-lithium ratio (Mg/Li) in solution from 40 to 2.1. This membrane was considered a low-cost and environmentally friendly new inorganic material. To enhance the kinetic diffusion of Li^+^ ions, He et al.^11^ prepared flakes based on a lithium/titanium/phosphate compound (LTP). The 2D layered structure exhibited a high aspect ratio and a richness of active sites, promoting a Li^+^ diffusion coefficient for the lamellar LTP anode of 3.12 × 10^–8^ cm^2^/s almost 2 orders of magnitude higher than that of the granular LTP anode. Guo et al.^12^ synthesized a 3D ionic conductor material based on a lithium/titanium/phosphorus compound named LTP via a solvothermal method followed by calcination in a nitrogen atmosphere. The authors reported that, for a specific sample, the initial discharge capacity reached up to 123.3 mAh·g^–1^ at a high rate of 10 C, highlighting its potential application as a cathode for lithium-ion batteries. Bol’shako*v* et al.^13^ synthesized a hierarchical structured material—nanotitanium-phosphorus double oxide (TPDO)—where the improved functionality was attributed to its multimodal porosity. The high dihydrophosphate content in TPDO indicated its potential as a proton conductor. A nanocomposite based on Fe, graphene oxide, and titanium phosphate was prepared. The new material was tested for photocatalytic degradation of the organic pollutant rhodamine B, with concomitant electricity generation. The degradation efficiency reached almost 80%.^14^ Magnesium represents the eighth most abundant inorganic element in the Earth’s crust.^15^ Magnesium as salts and/or oxides are interactive with other matters promoting multifunctionality. Kaushik et al.^16^ investigated how the doping of Ni/Zn/ferrite compound with magnesium affected its microwave absorption. It was highlighted that the addition of magnesium on the precursor structure generated a very efficient absorber, with an absorption capacity around 99% at 16.68 GHz. Lei et al.^17^ studied codoping of Sn–Mg in barium M-type hexaferrites (BaFe_12_O_19_) aiming to enable miniaturization for application in communication devices. The presence of Sn^4+^ and Mg^2+^ improved sample density and revealed excellent magnetic properties. Xie et al.^18^ modified wollastonite, doping it with dilute solution of magnesium nitrate. In this procedure, around 10% of calcium was substituted by magnesium. It was registered improvement of mechanical properties suggesting its application in bone defect repair. Tricalcium phosphate (TCP) was modified by doping with magnesium (Mg) and zinc (Zn). The influence on physical, mechanical, and biological properties was evaluated. According to Xue and collaborators, high mechanical strength, low resorption, and cell–material interaction candidates it for application in orthopedics and dentistry fields.^19^ Methylammonium lead iodide [CH_3_NH_3_PbI_3_], namely MAPbI3 perovskite, was modified by doping with magnesium iodide (MgI_2_). It was revealed that crystal grain size was increased and that the performance of the solar cell was improved.^20^ Wang et al.^21^ revealed that a high-performance kesterite thin film has been developed for use in solar cells after doping with magnesium salt. The current work aimed to study the effect of the incorporation of MgSO_4_ inside α-TiP with and without intercalation with ethylamine aqueous solution. Dry handling mixing and solvent-based methods routes were used. Structural, thermal, morphological, molecular relaxation, and electrochemical impedance spectroscopy techniques were used for characterization. It was affirmed that ethylamine aqueous solution had a crucial role as an intercalator of phosphate, modulating its properties.

## Experimental Section

### Material

Titanium isopropoxide (C_12_H_28_O_4_Ti), phosphoric acid (H_3_PO_4_, 85% w/w), ethanol (C_2_H_6_O, 99%), ethylamine aqueous solution (C_2_H_5_NH_2_, 70%, solution density = 0.81 g.mL^–1^) were all supplied by Sigma-Aldrich Ltd. Anhydrous magnesium sulfate (molecular mass = 120.37 g·mol^–1^, density = 2.66 g.mL^–1^) was purchase from Grupo Química, Brazil.

### α-TiP Synthesis

Following the methodology reported by Albitres et al.,^22^ synthesis of α-TiP was conducted considering the Ti/P molar ratio of 1:8. Under vigorous stirring, titanium isopropoxide (20 mL) was added dropwise to the phosphoric acid (35 mL) following the addition of 10 mL of deionized water. The reaction medium was kept at 120 °C, under reflux, for 24 h. Residual acid was released by washing with deionized water, using cellulose membrane dialysis bags until reaching pH = 6. The precipitate was dried at 90 °C until constant weight, being labeled as TiP.

### Intercalation of the αTiP with Ethylamine and Addition of Anhydrous Magnesium Sulfate

The intercalation of TiP with ethylamine and subsequent addition of magnesium sulfate was performed based on the article published by Albitres et al.^22^ α-TiP intercalation with ethylamine (E) was aimed to increase its lamellar spacing. The intercalation was carried out by dropping an ethanolic solution of ethylamine onto an ethanolic dispersion of α-TiP (molar ratio - E/αTiP = 2:1, 30̊C, stirring, 24 h). At the end, the dispersion was dried at 90 °C until constant weight. The intercalated sample was labeled as ETiP. For addition of MgSO_4_, two routes were experienced. At the first one, an ethanolic solution of MgSO_4_ (0.5 g of salt dissolved in 20 mL of ethanol) was dripped onto an ethanolic dispersion of ETiP (1 g of α-TiP in 10 mL of ethanol) at 30 °C under stirring, for 24 h. Afterward, the reaction medium was filtered and dried in an oven until constant weight. The dried product was labeled ETiP/OHMgSO_4_. At the second one, a solution of MgSO_4_ in ethylamine (0.1 g of salt dissolved in 4 mL of ethylamine) was prepared. The salt solution was dripped onto an ethylamine dispersion of ETiP (1 g of α-TiP in 10 mL of ethylamine) at 30 °C under stirring, for 24 h. Subsequently, the dispersion was filtered and dried at 90 °C, until constant weight. This product was named ETiP/EMgSO_4_. Finally, a dry handling mixing (preparation without any solvent) of α-TiP and MgSO_4_ was prepared, being labeled as αTiP/MgSO_4_(MF).

### Wide-Angle X-ray Diffraction

Wide-angle X-ray diffraction (WAXD) experiments were performed in a Rigaku Ultima IV diffractometer, with CuKα radiation (λ = 1.5418 Å) and experimental conditions of 40 kV, 20 mA, step of 0.05, ranging the 2θ angle from 2° to 50°.

### Thermogravimetry

Thermogravimetry was performed in TA Instruments model Q500, between 30 and 700 °C, at 10 °C.min^–1,^ and nitrogen as the carrier gas. Mass loss and derivative curves were obtained and the degradation steps and temperatures were determined.

### Fourier Transform Infrared Spectroscopy

Infrared spectroscopy was performed by using a PerkinElmer Frontier spectrometer. The spectra were taken from a KBr disk, in the range from 4000 to 400 cm^–1^, with 60 scans and 4 cm^–1^ of resolution.

### Scanning Electron Microscopy/Energy-Dispersive X-ray Spectroscopy

SEM images of the samples were captured using a TESCAN MIRA LMU scanning electron microscope (LowVac Mode UniVac) operated at 10 kV. The samples were coated with a thin layer of gold before imaging. Elemental analysis was performed using energy-dispersive X-ray spectroscopy (EDS) as an equipment accessory.

### Raman Spectroscopy

Raman spectroscopy was performed using a Jobin Yvon (Horiba Group) LABRAM system with a 632.8 nm He–Ne laser at low power (∼15 mW), coupled to an OLYMPUS BX40 polarized microscope. The spectral range was 3500 to 200 cm^–1^.

### Time-Domain Nuclear Magnetic Resonance (TD-NMR)

To assess the molecular dynamics of the materials, relaxometry measurements of nuclear spin relaxation times—spin-lattice and spin-spin—were evaluated. The technique was conducted in a Maran Ultra spectrometer (Oxford Instruments) with an 18 mm magnet bore, operating at 0.54 T (23.4 MHz for 1H). Spin-lattice relaxation time (T1H) was performed by inversion-recovery pulse sequence (recycle delay −180°, τ—90° acquisition data), 90° pulse of 7.5 μs, automatically calibrated by equipment software. Spin-spin relaxation time (T_2_H) was evaluated by using Magic Sandwich Eco (MSE) with the pulse sequence mentioned above. FID amplitude was considered for 2048 τ data points, interval of 1 μs, 16 scans, and 3 s of recycle and receptor gain of 4%. For both, the data were processed in commercial software, namely WinFit version 2.4.0.0 and Origin version 8.5.^23^

### Electrochemical Impedance Spectroscopy

Electrochemical impedance spectroscopy was performed using a Keithley Instruments 4200 Semiconductor Characterization System (4200-SCS USA) over a frequency ranging from 1 Hz to 1 MHz, at room temperature, as published by Arias et al.^24^ The AC and DC conductivities were evaluated. Equivalent electrical circuits were built based on Nyquist and Bode diagrams and by a phase diagram with the aid of a software program Nova 2.1.7 (METHOM AUTOLAB B.V.). Two replicates were evaluated.

## Results and Discussion

### Wide-Angle X-rays Diffraction

Figure 1 presents the diffraction patterns of the samples. αTiP shows diffraction peaks around 11.7°, 20.9°, 22.0°, 25.8°, and 36°, which are in accordance to results previously published elsewhere.^22,25^ Diffraction peaks at 18.4°, 24.6°, 25.0°, 26.4°, 27.8°, and 34.5° were observed for MgSO_4_, consistent with other reports.^26,27^ After modification with ethylamine aqueous solution, the ETiP sample showed that the original αTiP diffraction peak at 11.7° (basal spacing around 7.6 Å) disappeared, and a new peak appeared at 6.4°, with basal spacing increased to 13.8 Å. Additionally, a low-intensity diffraction peak appeared around 24–26°. This indicates that the intercalation of ethylamine into the αTiP galleries promoted partial enlargement of its original interlamellar spacing through a Bronsted-Lowry reaction between the NH_2_ group and the P–O–H group, resulting in the ionic species P–O– _3_HN^+^–CH_2_–CH_3_.^7^ García-Glez et al.^28,29^ found a similar result in their investigation on exfoliation and europium­(III) functionalization of αTiP with propylamine. Airoldi and Oliveira proposed a possible mechanism of TiP intercalation with alkylamines (methylamine to butylamine) in the presence of H_2_O and 1,2-dichloroethane (DCE), based on the amine protonation by P–OH groups and its insertion among the sheets. The study based on thermochemical cycle uses enthalpy values at each step of the proposed mechanism. Three steps were considered: dissociation of P–OH, O_3_P–OH = O_3_P–O^–^ + ^+^H, protonation of R–NH_2_ + H^+^ = H_3_^+^N–R, and formation of organic salt O_3_P–O^–^ + H_3_^+^N–R = O_3_P–O– H_3_^+^N–R. The solvation of the amine groups in the liquid medium seems to have an important role in the its interaction with the P–OH acid group on the matrix.^30^ When ETiP was modified with MgSO_4_ by using absolute ethanol as the liquid medium (ETiP/OHMgSO_4_), the diffraction peak at 6.3° was maintained, but a new peak appeared at 7.2°, which may indicate that a partial expulsion of free ethylamine molecules occurred from the phosphate interlamellar spacing owing to the entrance of magnesium salt. When ethylamine aqueous solution was also used as the solvent medium during the modification of ETiP with MgSO_4_ (ETiP/EMgSO_4_), no variation in the crystallographic profile was observed. The dry handling mixing [αTiP/MgSO_4_(MF)] showed the original diffraction angles of αTiP and MgSO_4_. XRD revealed that the intercalation with ethylamine aqueous solution had an important role in the incorporation of magnesium salt. It was possible to deduce that for the samples obtained by liquid medium, the magnesium salt is dissociated owing to the presence of water in both ethanol and ethylamine. On the contrary, for the dry handling mixing sample, the phosphate and the magnesium salt maintained their structural integrity.

**Figure 1:**
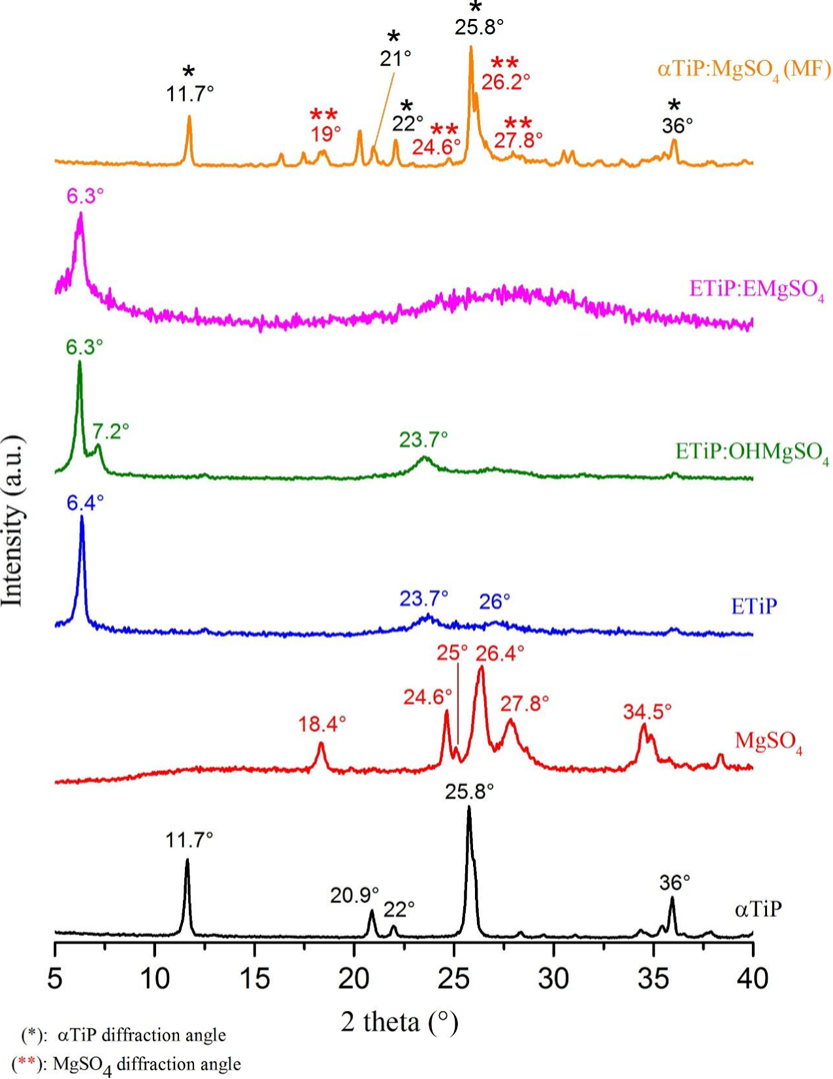
WAXD of αTiP, MgSO_4_, ETiP, ETiP/OHMgSO_4_, ETiP/EMgSO_4_, and αTiP/MgSO_4_(MF).

### Field Emission Scanning Electron Microscopy/Energy-Dispersive X-ray Spectroscopy

Figure 2a–f depicts the SEM images of the samples. In Figure 2a, αTiP displays stacks of hexagonal entities with heterogeneity in length and thickness.^5^ MgSO_4_ presented in Figure 2b has discontinuous clusters with no sharp geometrical shape. For ETiP (Figure 2c), it was possible to see that the thickness of the αTiP lamellae was higher, indicating the effect of ethylamine as an intercalator like reported by Garcia et al.^7^ For ETiP/OHMgSO_4_(Figure 2d), the presence of MgSO_4_ dissipated part of the ethylamine cloud, and the ETiP entities appeared more distinct. For ETiP/EMgSO_4_ (Figure 2e), ETiP structures were observed as embedded in a cloud of MgSO_4_ probably owing to the magnesium salt dissociation by the presence of water in the ethylamine. In the case of αTiP/MgSO_4_(MF) (Figure 2f), the image showed that the dry handling mixing result in physical agglomeration of both.

**Figure 2:**
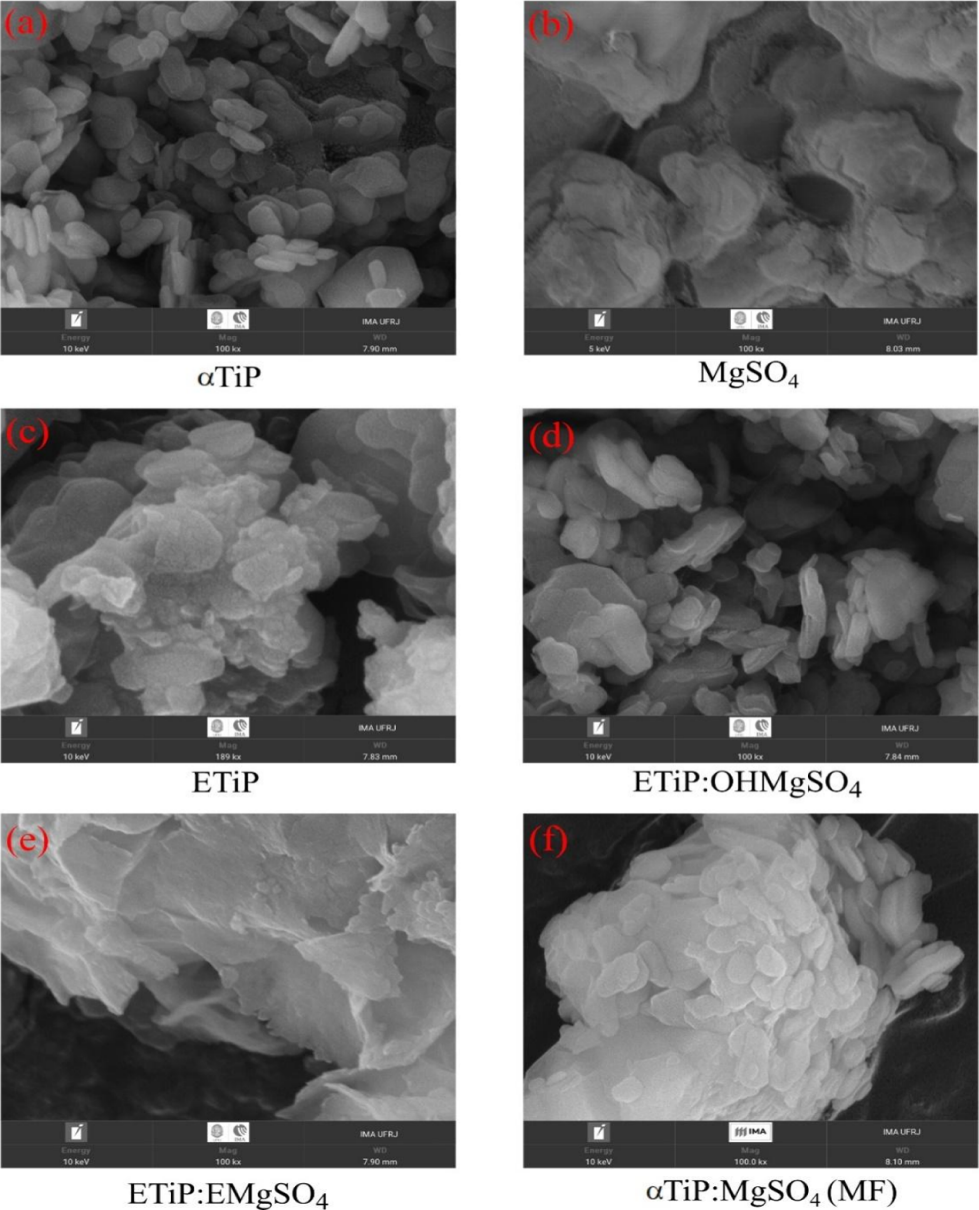
SEM images of (a) αTiP, (b) MgSO_4_, (c) ETiP, (d) ETiP/OHMgSO_4_, (e) ETiP/EMgSO_4_, and (f) αTiP/MgSO_4_(MF).

Figure 3 presents the spectra of the elemental analysis by EDS. The energy peaks corresponding to the K_α_ lines of Ti (4.5 keV), P (2.0 keV), Mg (1.2 keV), S (2.3 keV), O (5.0 keV), and N (0.39 keV) were detected.^31^ Figure 4 displays the elements’ relative percentages for each sample. It is important to point out that the carbon content of the support used in the sample detection was considered in the analysis. Therefore, the analysis could be considered semiquantitative. The absence of magnesium in the sample obtained by ethanol as liquid medium was observed. Its absence could be justified by differences in sensitivity to the technique—for example, oxygen is very sensitive and magnesium is less sensitive—and still its lower salt solubility in ethanol when compared to water. In favor, it could register an increase in oxygen content.

**Figure 3:**
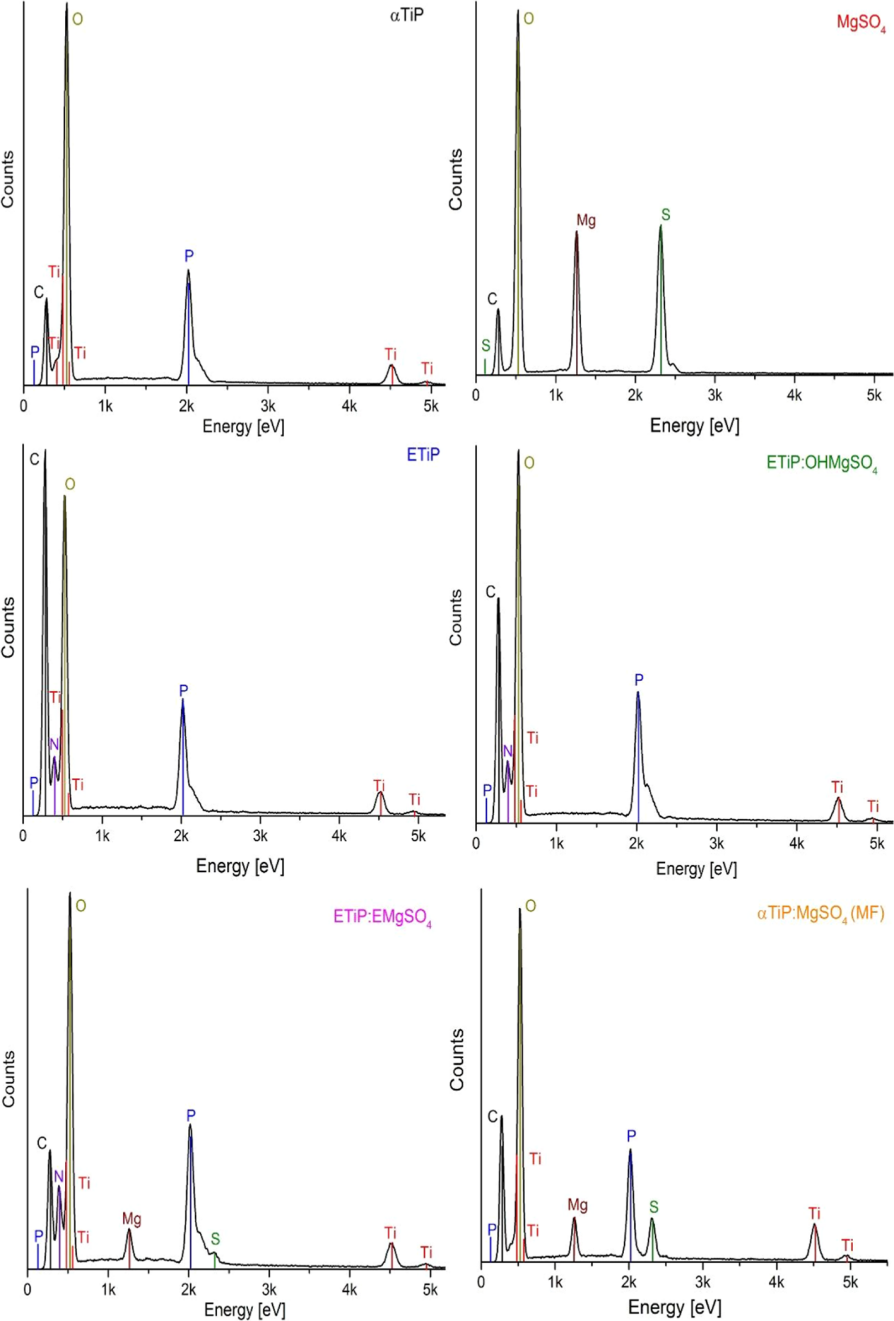
EDS of αTiP, MgSO_4_, ETiP, ETiP/OHMgSO_4_, ETiP/EMgSO_4_, and αTiP/MgSO_4_(MF).

**Figure 4:**
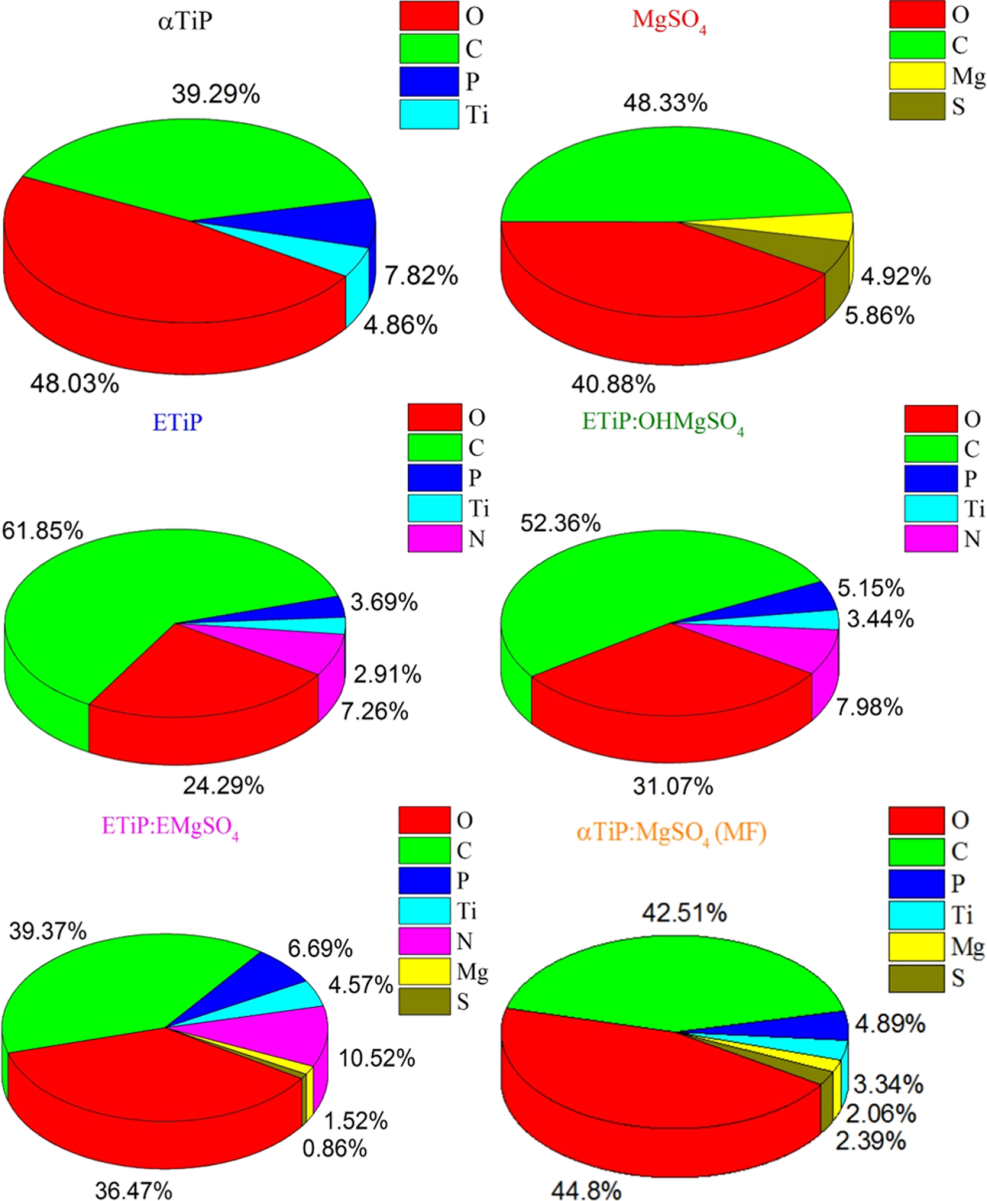
Relative percentages of the elements by EDS.

Figure 5 shows the elemental distribution and dispersion supported by SEM-EDS images. In the case of αTiP, both titanium and oxygen were well dispersed and evenly distributed throughout the sample, with oxygen being the most abundant element. The bright image reflects the crystalline nature of the phosphate. Regarding MgSO_4_, the SEM image depicts a crystalline material interspersed among amorphous regions, indicating an uneven distribution and areas without the inorganic salt. The predominance of oxygen was also noted. The ETiP image reveals a crystalline material with poorer elemental distribution and dispersion compared to the precursor αTiP, evidencing the effect of ethylamine incorporation. For αTiP/MgSO_4_(MF), it was possible to infer that the elements are unevenly distributed and dispersed similarly to notice in the SEM image. For ETiP/OHMgSO_4_ and ETiP/EMgSO_4_, the comparison between different solvent medium suggests that ethylamine aqueous solution favored the incorporation of MgSO_4_.

**Figure 5:**
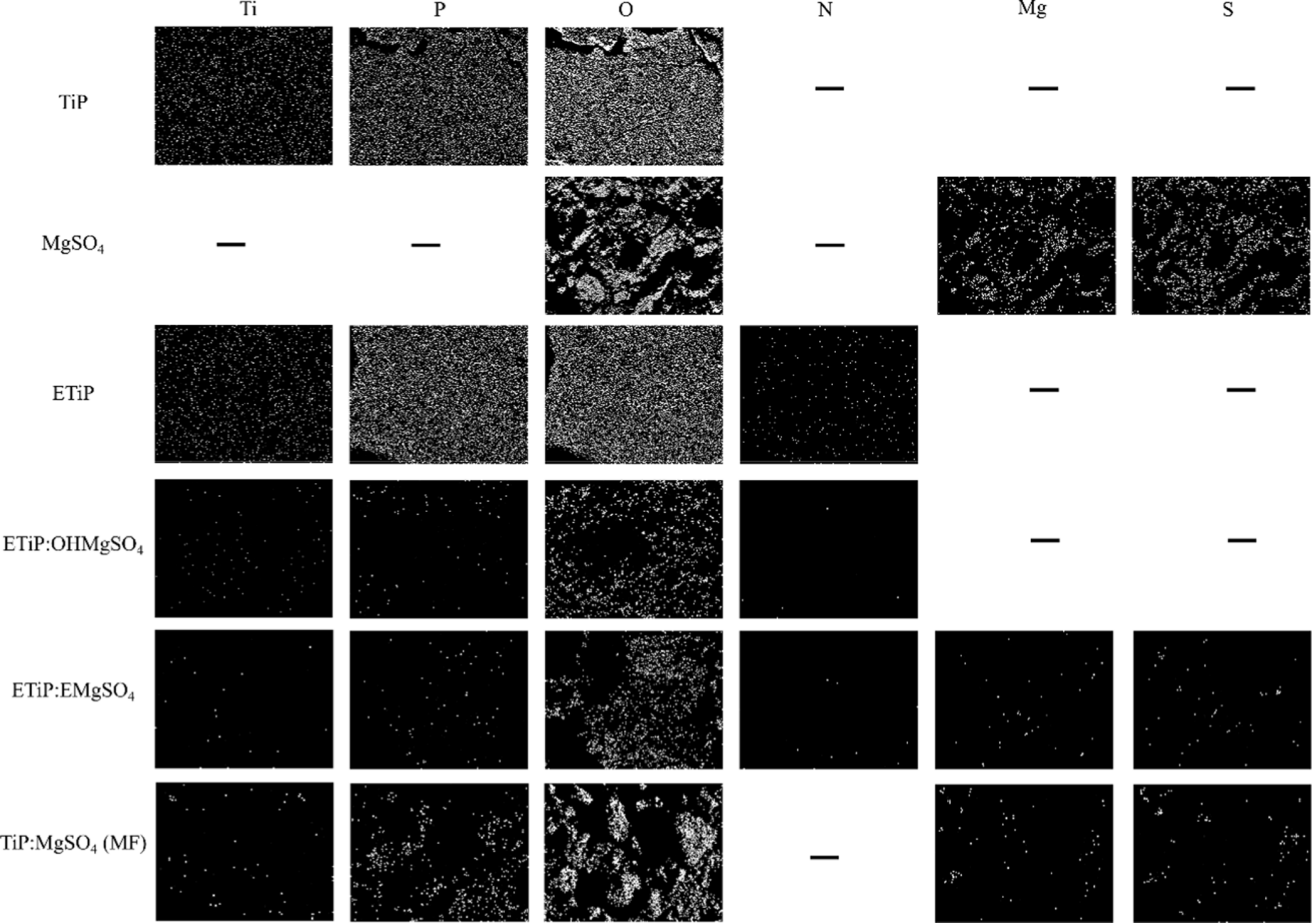
EDS elements distribution and dispersion for αTiP, MgSO_4_, ETiP, ETiP/OHMgSO_4_, ETiP/EMgSO_4_ e αTiP/MgSO_4_(MF).

### Thermogravimetry

Figure 6 shows the mass loss (TG) derivative thermogravimetric (DTG) curves. αTiP and MgSO_4_ exhibited two main degradation steps followed by ETiP/OHMgSO_4_, ETiP, αTiP/MgSO_4_(MF), and ETiP/EMgSO_4_. Table 1 summarizes the degradation steps. αTiP exhibited three distinct degradation steps: the first, between 100 and 150 °C, was attributed to the release of adsorbed water; the second, from 150–300 °C, corresponded to water loss from the crystalline lattice; and the third, from approximately 400–600 °C, was associated with the thermal transformation of phosphate into pyrophosphate corroborated by publication of Ortiz et al., Sahu & Parida and Garcia-Granda et al.^25,32,33^ Based on TGA data, the chemical formula of the synthesized titanium phosphate was determined as Ti­(HPO_4_)_2_ · 1.09H_2_O, corresponding to the α-TiP phase in agreement with Amghouz et al., Albitres et al., Bortun et al., and Szirtes et al.^5,22,34,35^ MgSO_4_ showed three degradation steps at 50–100 °C, 100–200 °C, and 250–375 °C, which were attributed to consecutive water loss as registered by Okhrimenko et al., Hongois et al. and Ma et al.^36–38^ For ETiP, three degradation stages were revealed in accordance to articles published by Albitres et al. and Garcia-Glez et al.^22,29^ The first one (30–100 °C) was attributed to the release of residual solvent or chemicals of synthesis and modification routes. The subsequent intervals between 100 and 230 °C and 240 and 400 °C were associated with the release of free and bonded amine molecules within the phosphate galleries, respectively, as reported in previous works by Albitres et al. and Mendes et al.^22,39^ The Bronsted-Lowry reaction between P–O–H groups in phosphate and _2_H–N groups induced both enlargement of interlayer spacing and blocking of the pyrophosphate formation evidenced by the absence of DTG peak around 400–500 °C. The same findings was highlighted by Albitres et al.^22^ in an investigation on intercalation of α-titanium phosphate with long-chain amine aided by short-chain amine and by Mapa et al.^40^ in which was studied sorption of organic dyes using Na_2_Ti­(PO_4_)_2_·H_2_O. Similarly to that found for ETiP, ETiP/EMgSO_4_ and ETiP/OHMgSO_4_ also presented three decays of degradation being one below 100 °C and the two other ones between 100 and 350 °C. The maximum degradation temperatures varied with the kind of liquid medium. In both, a degradation peak over 400 °C was noticed with higher intensity for ETiP/EMgSO_4_. Espina et al. monitored the mass loss of alkylamine (methylamine to hexylamine) after its intercalation inside α-TiP—addition of amine through atmosphere saturated with α-alkylamine vapor at room temperature. Depending on the alkylamine carbon number, the half of amine molecules are released at three temperature intervals, namely (115–150 °C), (180–205 °C) and 215–245 °C). The rest of the molecules were released at 300–340 °C and 350–370 °C.^41^ A careful examination of the thermogravimetric curves indicated that after the intercalation of α-TiP with ethylamine, the intercalated sample ETiP did not present the chemical transformation from phosphate to pyrophosphate. The increase in lamellar spacing blocks this transformation, as reported elsewhere.^22,40^ When ETiP was intercalated with magnesium salt, in solvent via, there was no change in lamellar spacing, and thus it would be expected that there would be no chemical transformation. On a careful examination of the TGA curve of α-TiP:MgSO_4_ it is possible to see a maximum at 451 °C representing the chemical transformation from phosphate to pyrophosphate. The derivative peak was less intense than TiP alone because the phosphate was mixing with MgSO_4_. Then, we emphasize that the derivative maximum observed in the ETiP/OHMgSO_4_ and ETiP/EMgSO_4_ samples is related to the presence of magnesium sulfate. For α-TiP/MgSO_4_, a degradation step below 100 °C was probably attributed to the release of water. The sequential degradation steps were ascribed to events of MgSO_4_ and chemical transformation of phosphate to pyrophosphate, respectively. Table 1 displays the mass loss for each degradation decay. The total mass loss was αTiP (12.5%), MgSO_4_ (18.0%), ETiP(32.3%), ETiP/OHMgSO_4_ (30.4%), ETiP/EMgSO_4_ (56.7%), and αTiP/MgSO_4_ (20.4%). The influence of ethylamine aqueous solution as a liquid medium during the insertion of magnesium salt was clearly exposed. The highest mass loss was achieved for ETiP/EMgSO_4_. With respect to ETiP/EMgSO_4_ and ETiP/OHMgSO_4_, we hypothesized that the degradation peak over 400 °C could be related to hydrogen bond formation between oxygen of sulfate groups in the magnesium salt and amine groups of free ethylamine molecules as schematically represented in Figure 7. In both samples, this mass loss stage was detected. Although by EDS the element magnesium was not detected in the ETiP/OHMgSO_4_, TGA confirms that the magnesium salt was admitted into the ETiP gallery in a smaller quantity. Summarizing, it was conjectured that the derivative peak at higher temperature for ETiP/EMgSO_4_ and ETiP/OHMgSO_4_ was related to interaction between sulfate groups and amine groups. This seems quite plausible since this step of degradation is very significant in the sample with a higher content of MgSO_4_ (ETiP/EMgSO_4_). Herein, the results confirmed that ethylamine aqueous solution played a key role on modulating phosphate properties and MgSO_4_ mobility within the phosphate matrix.

**Figure 6:**
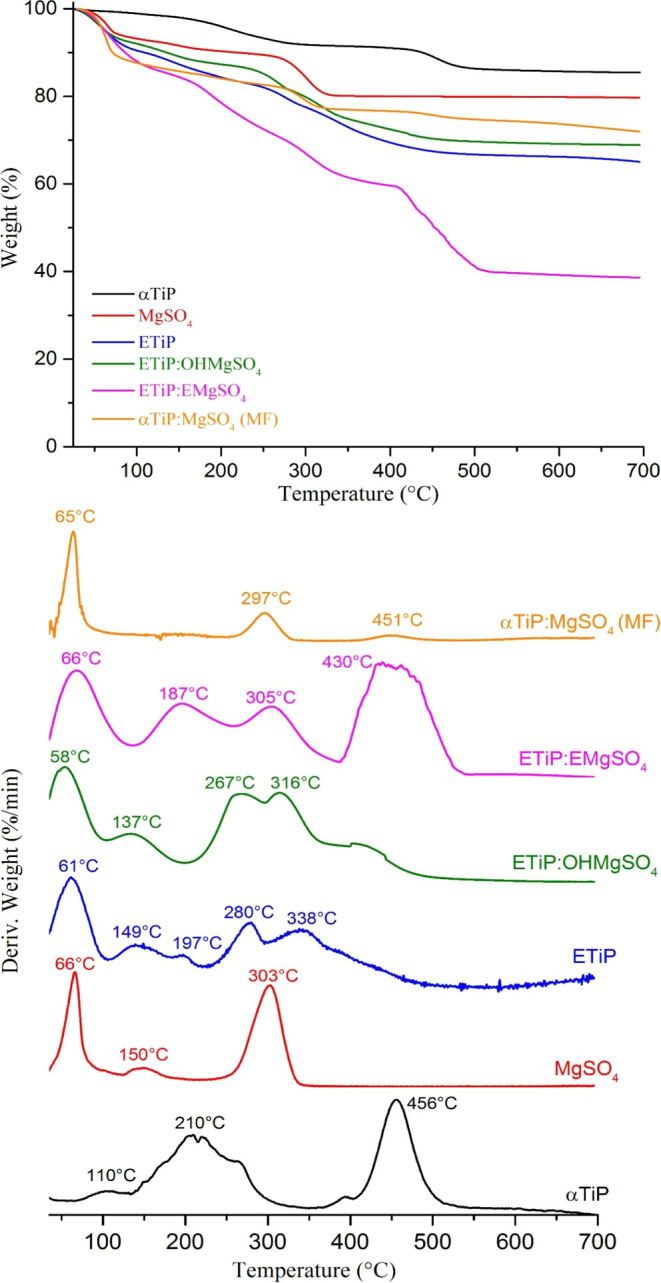
Mass loss and derivative curves for αTiP, MgSO_4_, ETiP, ETiP/OHMgSO_4_, ETiP/EMgSO_4_, and αTiP/MgSO_4_(MF).

**Figure 7:**
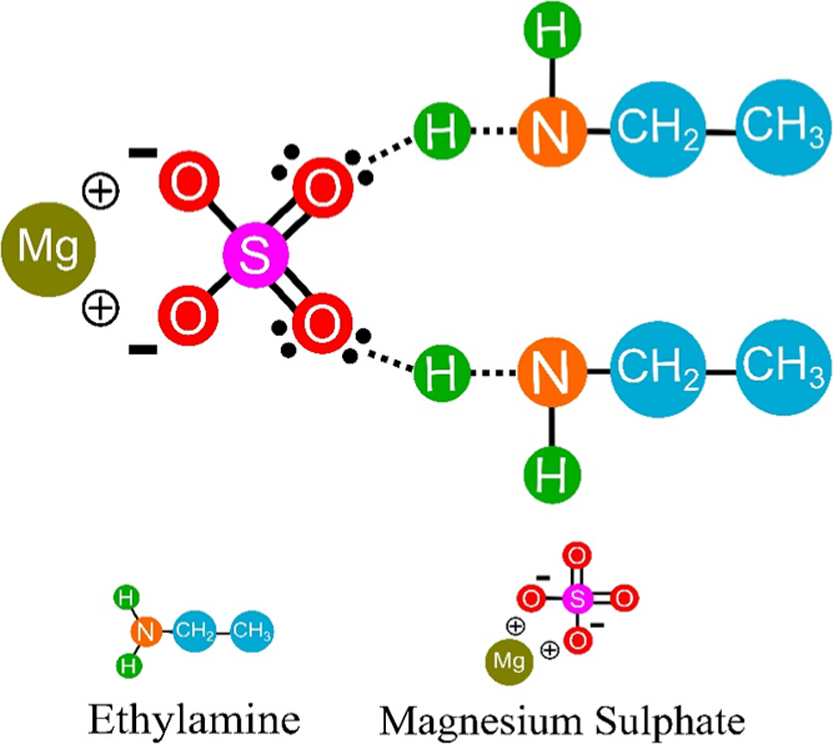
Supposed chemical interaction between magnesium sulfate and free ethylamine molecules inside ETiP galleries.

**Table 1:** Mass Loss and Derivatives Thermogravimetric Data

sample	first degradation step (°C)	mass loss (%)	second degradation step (°C)	mass loss (%)	third degradation step (°C)	mass loss (%)
αTiP	100–150	1	150–300	6.2	400–600	5.3
MgSO_4_	50–100	5.7	100–200	2.9	250–375	9.4
ETiP	<100	9.4	100–220	7.0	220–450	15.9
ETiP/OHMgSO4	<100	8.0	100–400	19.6	400–500	2.8
ETiP/EMgSO4	<100	12.0	150–400	24.8	400–550	19.9
αTiP/MgSO_4_(MF)	<100	12.4	250–350	5.7	400–550	2.3

### Fourier Transform Infrared Spectroscopy

Figure 8 shows the infrared spectra. Concisely, Table 2 summarizes the most relevant absorption and their attributions. αTiP exhibited absorptions bands at 3,566, 3,479, and 3017 cm^–1^ which are associated to the hydrogen bond between P–O–H groups and near 1620 cm^–1^ related to the hydrogen bond of water confined within its crystalline region as reported by Guo et al., Bruque et al., Pipi & Carmo and Alam et al.^2,42–44^ Absorption related to P–O–H, P–O, P–O–P, and PO_4_^2–^ were depicted around 1250; 1122; 1032; 1009; 971, and 721 cm^–1^ and Ti–O–P was observed at 615 cm^–1^ as registered by Sahu et al., Alam et al., Takahashi et al., and Guo & Han.^31,44–46^ For MgSO_4_, a broad absorption around 3700–3000 cm^–1^ - hydrogen bonding of water molecules—besides 1115 and 617 cm^–1^—SO_4_^2–^ vibrations—were registered as published by Jini et al.^26^ For ETiP, the absorptions at 3566 and 3479 cm^–1^ disappeared, being replaced by a broad and intense absorption. New absorptions at 3000–2700 cm^–1^ appeared, which were attributed to C–H vibrations of CH_2_ and CH_3_ groups of ethylamine molecules. The absorption around 1570–1520 cm^–1^ appeared owing to the Bronsted-Lowry reaction between the NH_2_ group and the P–O–H group, resulting in the ionic species P–O^–+^_3_HN–CH_2_–CH_3_, which promoted the enlargement of αTiP d_spacing_. These findings were endorsed by articles published by Albitres et al., Garcia-Glez et al. and Airoldi & Oliveira.^22,28,29,47^ Changes between 1300 and 800 cm^–1^ were ascribed to the presence of ethylamine inside αTiP crystalline arrangement as reported by Mendes et al. and Bestaoui et al.^39,48^ The Ti–O–P–O–H absorption rendered broadened and it was shifted to a higher wavenumber as registered by Garcia et al. and Albitres et al.^7–22^ The spectrum of αTiP/MgSO_4_(MF) evidenced the original absorptions of each precursor. The spectra of ETiP/EMgSO_4_ and ETiP/OHMgSO_4_ showed similarity but some difficulty was found to sign specific distinction among absorptions of P–O–H; P–O; P–O_4_^2+^ and SO_4_^2–^ linkages in the spectral region at 1300–800 cm^–1^. Herein, the X-ray diffraction indicated that magnesium salt is dissociated inside the ETiP galleries while TGA evidenced the chemical interaction between magnesium salt and ethylamine molecules. Based on these premises, we proposed to evaluate the efficiency of the liquid medium in the intercalation of magnesium salt by quantification of the peak area (absorbance mode) in the interval at 1300–800 cm^–1^. The peak area showed the following order: 274 > 219 > 143 u^2^, for ETiP/EMgSO_4_, ETiP/OHMgSO_4,_ and ETiP, respectively. The result confirms that ethylamine was the most efficient solvent medium and corroborates the X-ray diffraction and thermogravimetry.

**Figure 8:**
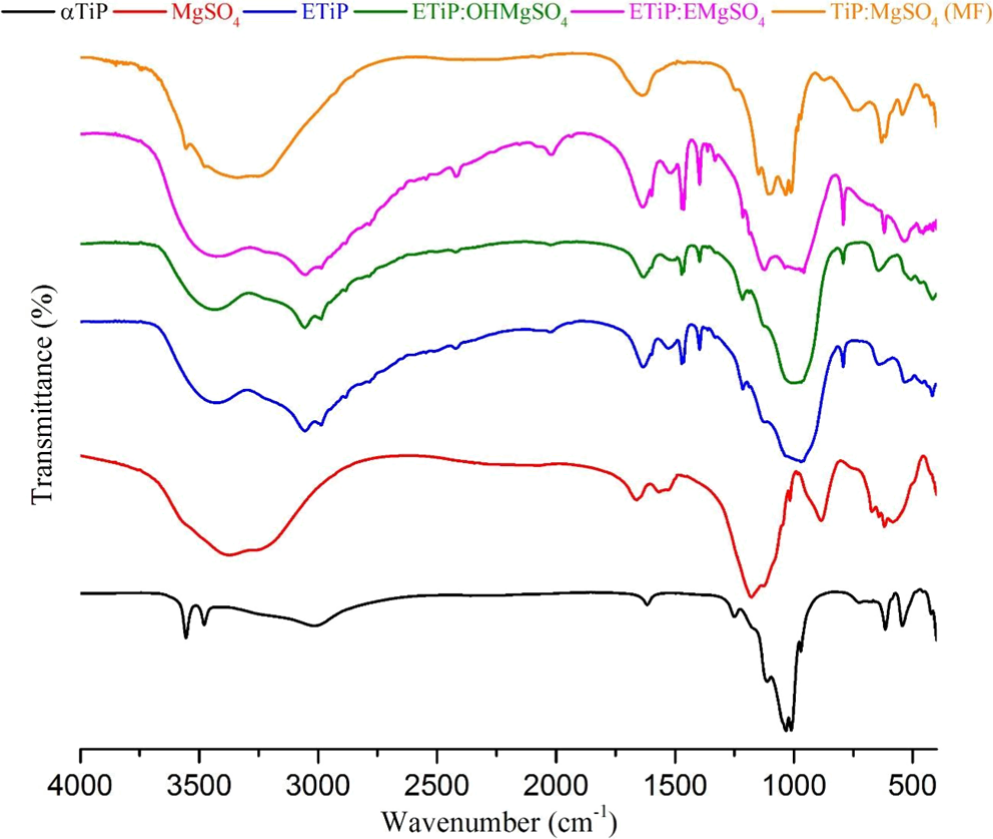
FTIR spectra of αTiP, MgSO_4_, ETiP, ETiP/OHMgSO_4_, ETiP/EMgSO_4_, and αTiP/MgSO_4_(MF).

**Table 2:** Samples’ Infrared Absorptions

sample	assignment (cm^–1^)	attribution
αTiP	3566 and 3017	P–O···H···O–H_2_ symmetric and asymmetric stretching
	3079 and 1620	P–O–H stretching and angular deformation
	1250; 1122; 1032; 1009	P–O–H; P–O; PO_4_^2+^ deformation and out-of-plane vibration
	971 and 721	P–O–P vibration
	615	Ti–O–P–O–H vibration
MgSO_4_	3700–3000	H–O–H stretching
	1115–617	SO_4_^2–^ vibrations
ETiP	3500	P–O–_3_H^+^N–CH_2_–CH_3_ vibration
	3020	P–O···H···O–H_2_ asymmetric stretching
	3000–2700	C–H stretching of CH_2_ and CH_3_ groups
	1570–1520	P–O–_3_H^+^N–CH_2_–CH_3_ asymmetric vibration
	619	Ti–O–P–O–H vibration
ETiP/OHMgSO_4_	3500	P–O–_3_H^+^N–CH_2_–CH_3_ vibration
	3020	P–O–H–O–H_2_ asymmetric stretching
	3000–2700	C–H stretching of CH_2_ and CH_3_ groups
	1570–1520	P–O–_3_H^+^N–CH_2_–CH_3_ asymmetric vibration
	619	Ti–O–P–O–H vibration
ETiP/EMgSO_4_	3500	P–O–_3_H^+^N–CH_2_–CH_3_ vibration
	3020	P–O···H···O–H_2_ asymmetric stretching
	3000–2700	C–H stretching of CH_2_ and CH_3_ groups
	1570–1520	P–O–_3_H^+^N–CH_2_–CH_3_ asymmetric vibration
	619	Ti–O–P–O–H vibration
αTiP/MgSO_4_(MF)	3566 and 3017	P–O···H···O–H_2_ vibration
	3079 and 1620	P–O···H···O–H_2_ asymmetric stretching
	1250; 1122; 1032; 1009	P–O–H; P–O; PO_4_^2+^ deformation and out-of-plane vibration
	971 and 721	P–O–P vibration asymmetric vibration
	615	Ti–O–P–O–H vibration
	3700–3000	H–O–H stretching
	1115–617	SO_4_^2–^ vibrations

### Raman Spectroscopy

Figure 9 displays the most significant Raman shifts. For αTiP, a vibrational mode at 1,008, cm^–1^ (symmetric stretching (ν1) of PO_4_^2–^) besides 424 and 324 cm^–1^ [PO_4_^2–^ twisting (ν2) and scissoring (δ) modes and interlamellar water molecules] were registered endorsed by articles published by Albitres et al., Schmultz et al., and Slade et al.^22,49,50^ For MgSO_4_, intense vibrational modes at 1045 and 1014 cm^–1^ [SO_4_^2–^ symmetric stretching (ν1)], a medium intensity one at 497 cm^–1^ [SO_4_^2–^ torsional mode (ν2)], and other ones at 1275; 1215 and 1151 cm^–1^[SO_4_^2–^asymmetric stretching of the S–O bond (ν3)] and at 693 and 618 cm^–1^ [SO_4_^2–^ bending mode (ν4)] were noticed. These assignment are in agreement with the reports by Wang et al. on a systematic Raman spectroscopic study of hydration states of magnesium sulfates.^51^ The most relevant vibrational modes of ETiP were C–N stretching [997, 875, and 734 cm^–1^], C–N–H bending [275 cm^–1^], CH_3_ asymmetric/symmetric stretching [2984 and 2888 cm^–1^], CH_2_ symmetric stretching [2948 cm^–1^], CH_3_ bending [1460 cm^–1^], CH_3_ asymmetric deformation [1154 cm^–1^], PO_4_^2–^ asymmetric and symmetric stretching [1296; 1063; 1008 and 997 cm^–1^]. These assignments are corroborated by studies of Albitres et al. and Rajini et al.^22,52^ For dry handling mixing [αTiP/MgSO_4_(MF)], the most important vibration modes of αTiP around 1008; 976; 424 and 324 cm^–1^ and MgSO_4_ (1151; 1045; 1,014, 693, and 618 cm^–1^) were preserved. ETiP/OHMgSO_4_ presented vibration modes at 1151; 1002; 945; 879; 450; and 339 cm^–1^ (high intensity) and at 739; 534; 200; and 127 cm^–1^ (low intensity) while ETiP/EMgSO_4_, showed intense vibrations at 976; 877; 661; 495; and 267 cm^–1^, with weaker peaks at 1144 and 1052 cm^–1^. Figure 10 shows all Raman shift spectra in the spectral region between 1500 and 200 cm^–1^ to better understand the effect of the ETiP intercalation with magnesium sulfate using ethanol and ethylamine aqueous solution as a liquid medium. ETiP showed an enlarged and low intense peak between 1014 and 976 cm^–1^ that appeared jointly with other ones at 875, 734, 560, and 496 cm^–1^. For ETiP/OHMgSO_4_, an enlarged peak appeared between 1045 and 875 cm^–1^ where defined maximum can be seen at 1014; 976 and 875 cm^–1^. Low intense peaks at 450 and 339 cm^–1^ were detected. This sample built via ethanol as liquid medium revealed some vibrations, which could be attributed to PO_4_^2–^ (1008 and 945 cm^–1^) and SO_4_^2–^ (1014 cm^–1^) besides the decrease of C–N vibration at 734 cm^–1^. Then, it is reasonable to infer that when the magnesium salt access ETiP galleries some free ethylamine molecules are expelled. This could explain the appearance of a new diffraction angle (7.2°) in the X-ray analysis. For ETiP/EMgSO_4,_ vibrations at 976 cm^–1^ (PO_4_^2–^) and 877 cm^–1^ (C–N) were associated with ETiP contribution while too low intense vibration at 1014 cm^–1^ was ascribed to MgSO_4_ meaning that magnesium salt is dissociated. The original vibrations at 739 cm^–1^ (C–N) and 693 and 618 cm^–1^ (SO_4_^2–^) rendered a new vibration at 661 cm^–1^. We can hypothesize that this vibration could have arisen owing to the chemical interaction between oxygen atoms of sulfate groups and amine groups through the hydrogen bond as highlighted in the thermogravimetric analysis. Figure 11 shows a speculative schematic representation of the αTiP after intercalation with ethylamine aqueous solution (ETiP) and its intercalation with magnesium sulfate in different liquid medium. The intercalation of αTiP with ethylamine aqueous solution provokes the increase of the lamellar spacing, formation of ionic species P–O^–+^_3_HN–CH_2_–CH_3_ attached to the phosphate structure and the presence of free ethylamine molecules into ETiP galleries. When the latter was submitted to intercalation with magnesium sulfate, the lamellar spacing and P–O– _3_HN^+^–CH_2_–CH_3_ were preserved but we believe that magnesium salt is predominantly dissociated in the sample ETiP/EMgSO_4_ owing to high aqueous content in the ethylamine aqueous solution, supporting the chemical interaction between sulfate groups and amine ones. The results are supported by DRX and TGA characterizations.

**Figure 9:**
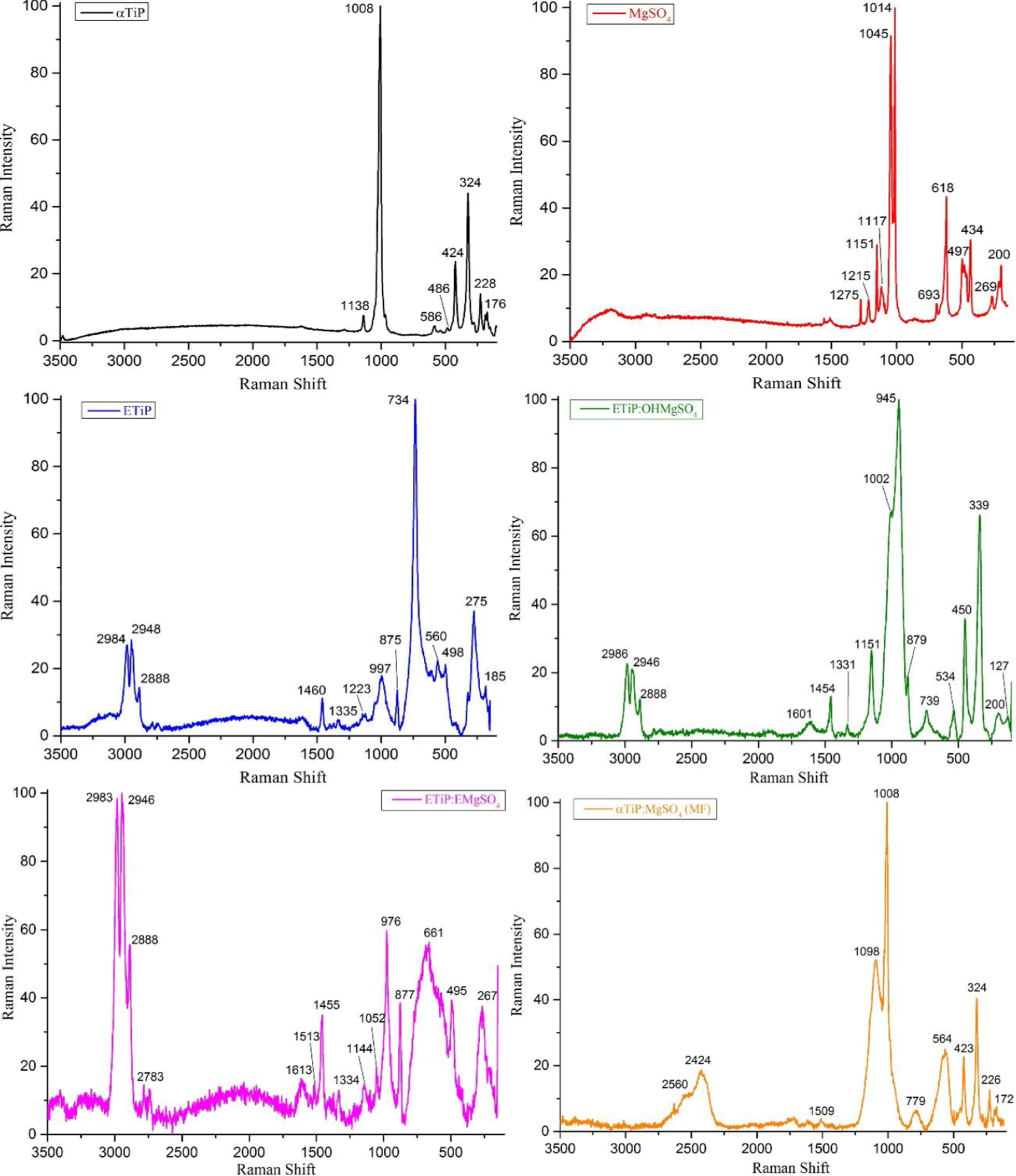
Raman shift of αTiP, MgSO_4_, ETiP, ETiP/OHMgSO_4_, and ETiP/EMgSO_4_ and αTiP/MgSO_4_(MF).

**Figure 10:**
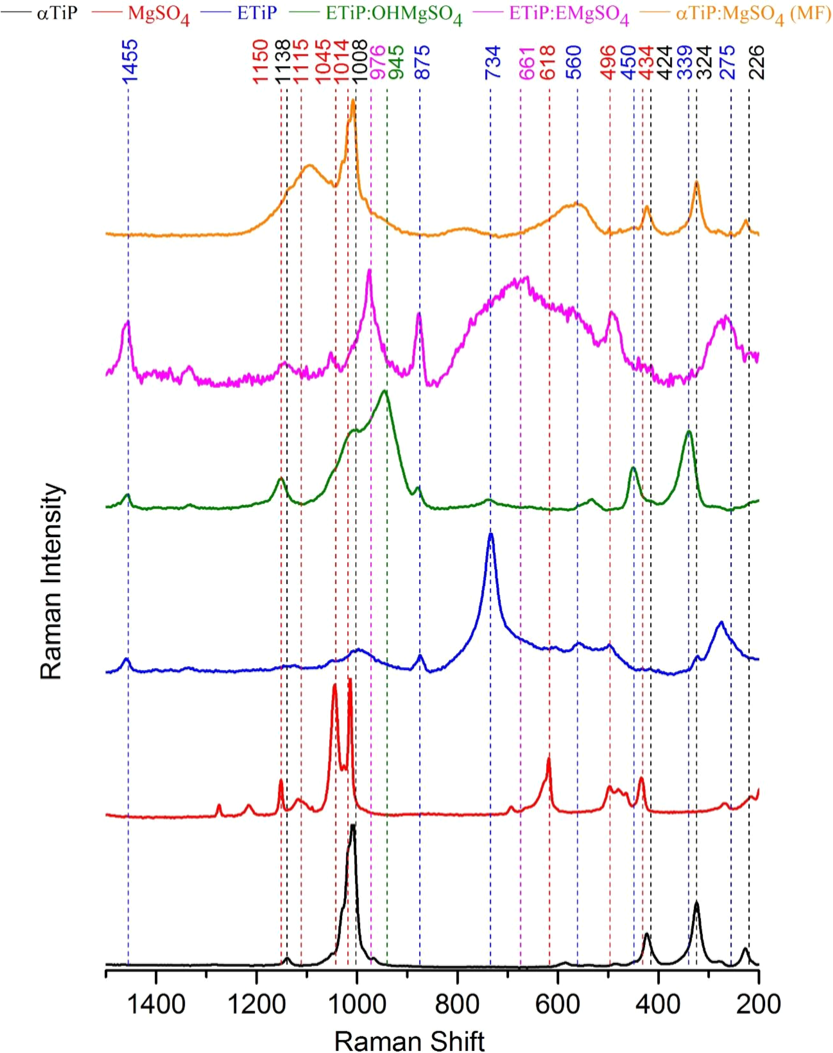
Evaluation of Raman shift at 1500–200 cm^–1^.

**Figure 11:**
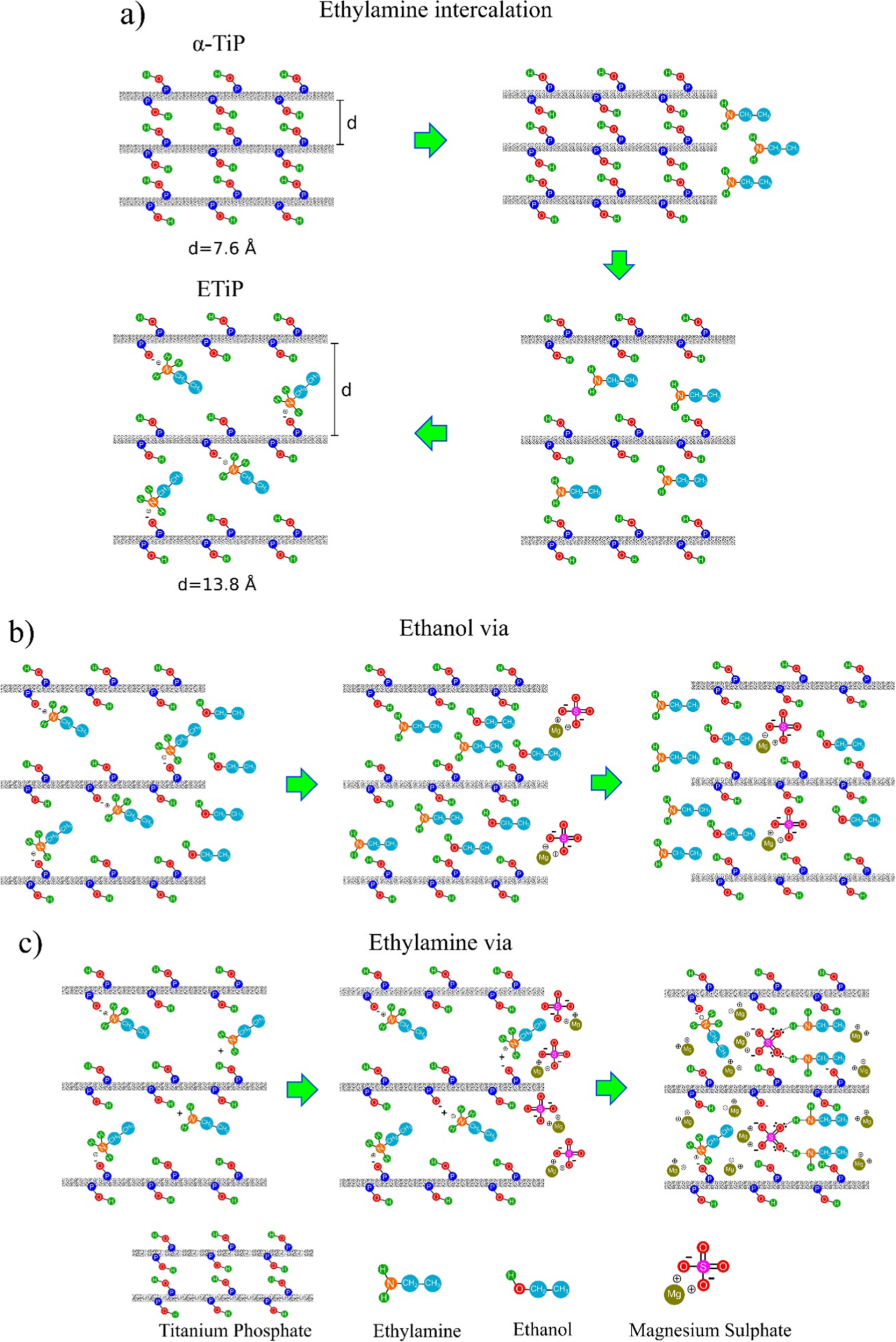
Speculative schematic representation of the αTiP modification: (a) after intercalation with ethylamine aqueous solution (ETiP); (b) ETiP after intercalation with magnesium sulfate being ethanol as the liquid medium; (c) ETiP after intercalation with magnesium sulfate being ethylamine aqueous solution as the liquid medium.

### TD-NMR

Time-domain nuclear magnetic resonance enables monitoring of how the hydrogen nucleus returns to its equilibrium state after being disturbed by an external magnetic field.

### Spin-Lattice Relaxation

Figure 12 depicts the domain distribution curves of spin-lattice relaxation (T_1_H). It is important to mention that owing to the absence of hydrogen atoms in its chemical structure, MgSO_4_ showed no relaxation curve. αTiP presents a unique relaxation domain in the range of 5–100 ms, shown as a broad peak with an adjacent shoulder. After modification with ethylamine, the original relaxation domain split into two distinct peaks: a low-intensity peak at 2–4 ms and a high-intensity one between 5 and 30 ms. Resembling domain curves were revealed for ETiP/OHMgSO_4_ and ETiP/EMgSO_4_, exhibiting a single domain between 3 and 40 ms. Two distinct domains at 1–10 ms and 4–200 ms were depicted for αTiP/MgSO_4_ (MF). Aguiar and Tavares studied composites containing polycaprolactone (PCL), clay, and vanadium oxide. Spin-lattice relaxation revealed that, besides homogeneity, the oxide improved distribution and dispersion due to intermolecular interactions with the PCL matrix, as evidenced by the narrowing of the domain curves.^53^

**Figure 12:**
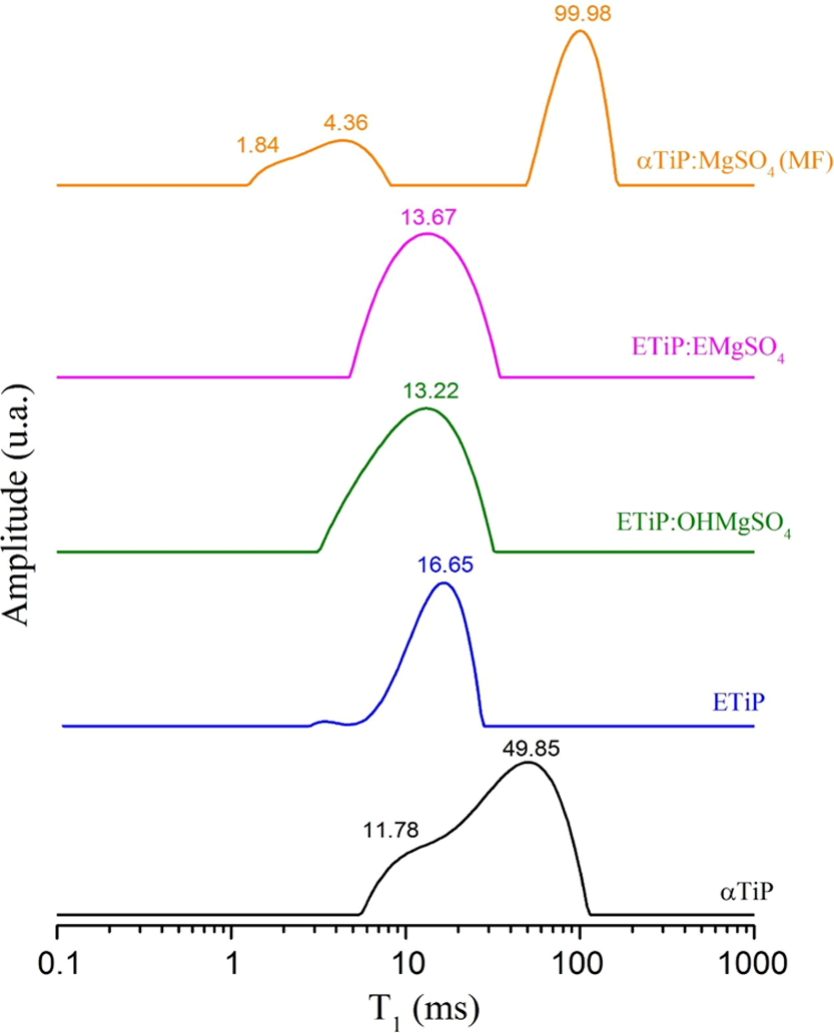
NMR spin-lattice domain curves of αTiP, ETiP, ETiP/OHMgSO_4_, ETiP/EMgSO_4_, αTiP/MgSO_4_ (MF).

### Spin-Spin Relaxation

Table 3 summarizes the evaluation of samples’ spin-spin relaxation. For samples where the MgSO_4_ was incorporated via solvent—ETiP/OHMgSO_4_ and ETiP/EMgSO_4_, a significant decrease in the rigid fraction and an expressive increase of T_2_H were noted. In contrast, for dry handling mixing [αTiP/MgSO_4_ (MF)], no change in the rigid fraction and only a very slight increase in T_2_H were noticed. Concerning the mobile fraction, a noticeable increase was detected in the solvent-mediated samples, whereas only an insignificant variation was observed for the dry-handling mixing sample. The solvent-mediated samples also exhibited a substantial increase in T_2_H. It is worth mentioning that only the samples containing ETiP displayed an intermediate fraction, which showed higher T_2_H values than those of the rigid fraction. Shape memory polymers are a category of polymers that, when incorporated into a polymer chain, provide a shape memory effect. D’hollander et al.^54^ studied the shape memory effect of polycaprolactone (PCL) as inserted in a multiblock polyurethane based on the condensation of 1,4-butanediol (BDO) with hexamethylene diisocyanate (HDI). From T_2_H measured at 70 °C, the higher the molar ratio of HDI, the greater the formation of the rigid fraction and, inversely, the lower the formation of the mobile fraction. It is clear that the ethylamine affects the relaxometric movement of phosphate when inserted into αTiP galleries, since the T_1_H and T_2_H shifted to lower and higher relaxation times, respectively. Even with the introduction of MgSO_4_ in ETiP, the T_1_H, and T_2_H were altered mostly for samples modified via solvent. The one labeled as dry handling mixing revealed high T_1_H and no significant variation in T_2_H. It was deduced that for the samples containing ethylamine, the relaxation process was driven by the amine. Farber et al.^55^ published a revision article highlighting the importance of porous materials. Pores ranging from angstroms to centimeters facilitate the transfer of multiple energy vectors, such as mass, charge, heat, radiation, and pressure. Controlling the structural hierarchy, textural porosity, and roughness are important parameters for developing efficient materials. The mesoporous structure of titanium phosphate was highlighted by Chowdhury and Naskar^56^ and Guo et al.^46^ concerning lead removal and hydrogen generation, respectively. We believe that probably the mesoporous structure of the phosphate could also have contributed for relaxation processes. Shortly, spin-lattice and spin-spin relaxations demonstrated that both ethylamine aqueous solution and magnesium sulfate had great influence on the molecular mobility of phosphate matter. The results are aligned with those evidenced by DRX, TGA, and Raman spectroscopy.

**Table 3:** NMR Spin-Spin Relaxation Data

sample	rigid fraction (%)	*T*_2*R_ (μs)	intermediate fraction (%)	*T*_2*I_ (μs)	mobile fraction (%)	*T*_2*M_ (μs)
αTiP	97.91	13.2	--	--	2.09	255.5
MgSO_4_	78.97	9.3	--	--	21.03	30.6
ETiP	26.97	15.0	31.18	65.7	41.85	342.7
ETiP/OHMgSO_4_	22.23	51.1	38.90	73.5	38.87	514.3
ETiP/EMgSO_4_	11.35	95.7	39.08	166.9	49.57	305.0
αTiP/MgSO_4_ (MF)	94.48	19.6	--	--	5.52	140.4

### Electrical Impedance Spectroscopy

Figure 13 shows the Nyquist diagrams, which were plotted to understand how each sample responds to the frequency. It was noticed that all samples exhibit a typical single capacitive semicircle in different frequencies. Concerning the size of semicircle radius, it was viewed that αTiP e MgSO_4_ presented higher value than those observed for ETiP, ETiP/OHMgSO_4_, ETiP/EMgSO_4_, and αTiP/MgSO_4_(MF) as indicative of the depression in the resistance of the electrical current flow. The more expressive result was ascribed to ETiP/EMgSO_4_. Bode diagrams of Z and phase angle as function of frequency were plotted as can be seen in Figure 14. Concerning the plot Z vs frequency (Figure 14a), αTiP revealed a smooth and continuous decrease of Z with frequency around 10^4^ Hz and after that an abrupt drop was noticed. MgSO_4_ and αTiP/MgSO_4_(MF) showed stability up to 4 × 10^2^ and 2 × 10^4^ Hz, respectively, but upper a continuous decrease occurred. Also, a continuous decrease was observed for ETiP and ETiP/OHMgSO_4_. ETiP/EMgSO_4_ revealed a continued decrease and the lowest values of Z with the frequency. For the phase angle plot (Figure 14b), except for the ETiP/EMgSO_4_, all other samples presented discontinuous curves. For the precursors and αTiP/MgSO_4_(MF), the phase angles showed higher values than the others, decreasing up to a certain frequency and right after increased. Discontinuity occurred around 10^2^ Hz and 10^3^ Hz for MgSO_4_, αTiP and αTiP/MgSO_4_(MF). The ETiP and ETiP/OHMgSO_4_ curves are superimposed showing discontinuity around 10^4^ Hz. Although the phase angle continuously decreases with frequency no discontinuity was noticed for ETiP/EMgSO_4_.

**Figure 13:**
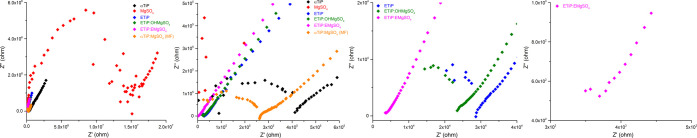
Nyquist diagrams of sample behavior as function of variation of Z″ versus Z′.

**Figure 14:**
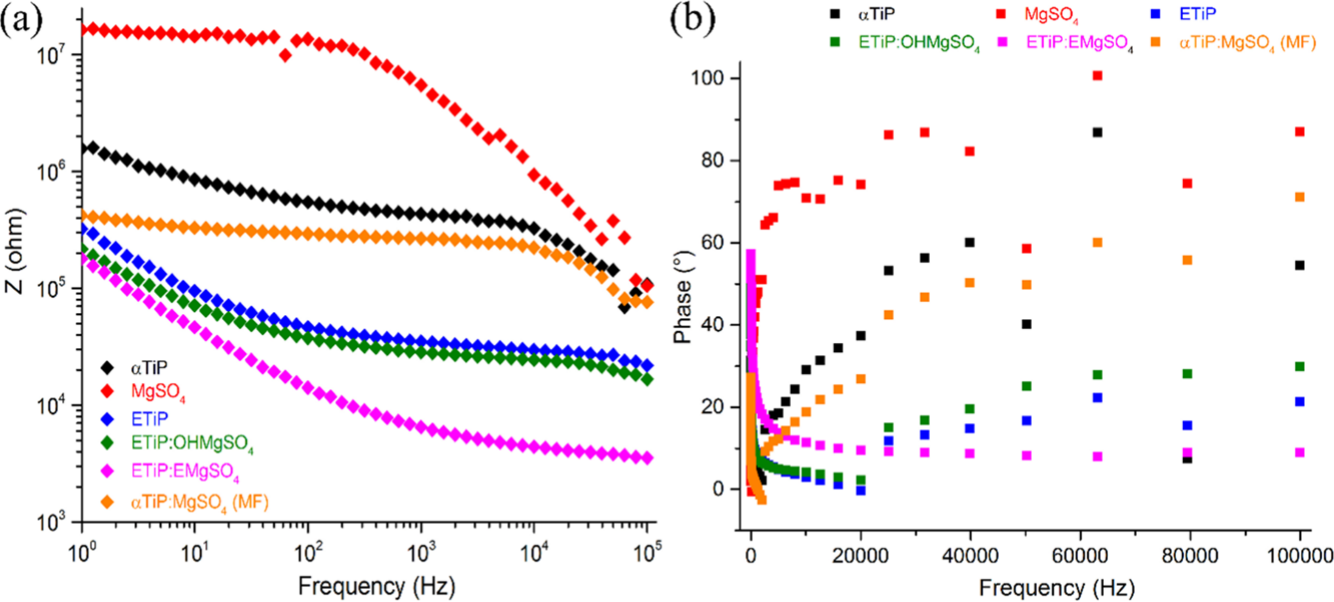
Bode diagrams: (a) Z vs frequency and (b) phase angle vs frequency.

DC current vs voltage plot (Figure 15) depicted no variation of current in the range of voltage for MgSO_4_, αTiP, and αTiP/MgSO_4_(MF). For ETiP and ETiP/OHMgSO_4_, the curves are overlapped and the variation of current occurred from 3 V. For the ETiP/EMgSO_4_, significant increase of current started around 2.5 V.

**Figure 15:**
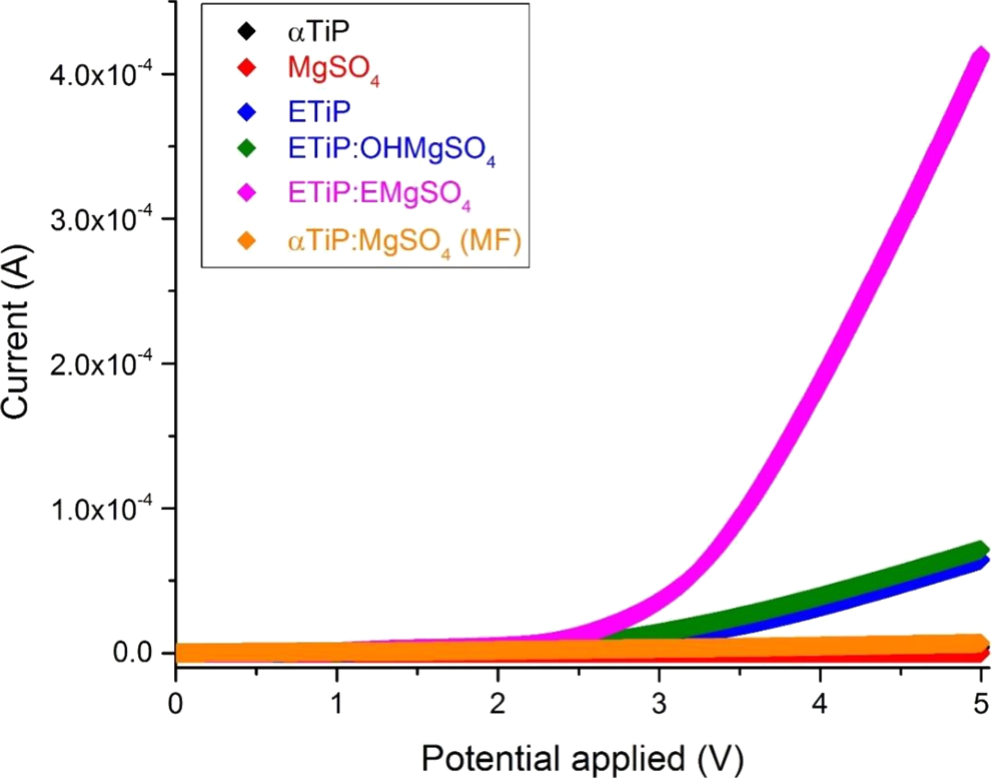
DC current vs voltage of αTiP, MgSO_4_, ETiP, ETiP/OHMgSO_4_, ETiP/EMgSO_4_, αTiP/MgSO_4_(MF).

From Nyquist and Bode diagrams it is possible to build models of an electrical equivalent circuit (EEC) with aiding computer program. The latter may be used to interpret experimental data. According to Ehrensberger and Gilbert,^57^ EEC are used to quantify the faradaic and nonfaradaic electrochemical processes at electrified interfaces. The Faradaic processes taking place at the interface are modeled as resistors and the separation of charge associated with non-Faradaic processes is modeled as a parallel-plate capacitor. Figure 16 presents the representative EEC for each sample. Depending on the electrical behavior, an EEC can be constituted by the following components: resistor (*R*), capacitor (*C*), Warburg impedance element (*W*), and a constant phase element (*Q*), associated in series or parallel. The EEC of the precursors αTiP and MgSO_4_ are different. When αTiP was intercalated with ethylamine aqueous solution a similar EEC was achieved. Independently on the liquid medium, the original EEC of ETiP was modified when magnesium salt was added. For ETiP/OHMgSO_4_ and ETiP/EMgSO_4_, the capacitor element (C) was replaced by a Warburg impedance element (W). By electrochemical impedance spectroscopy, Churikov et al.^58^ studied the behavior the reversible lithium intercalation from nonaqueous electrolyte into Sn films. The impedance spectra of lithium-tin (LixSn) electrodes have a complicated shape and different EEC were built depending on the electrode state.

**Figure 16:**
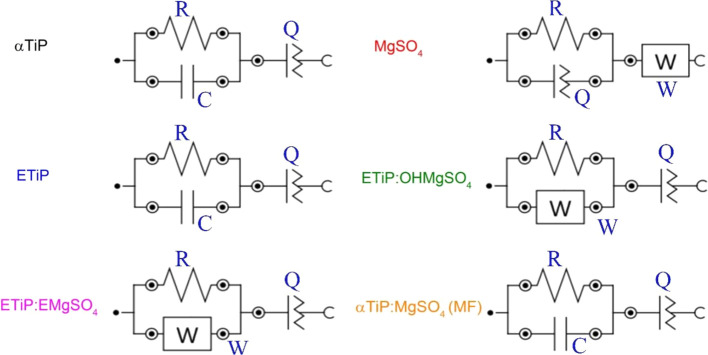
Equivalent electrical circuit of αTiP, MgSO_4_, ETiP, ETiP/OHMgSO_4_, ETiP/EMgSO_4_, αTiP/MgSO_4_(MF).

Equivalent circuits modeling of the impedance spectra of LixSn electrodes after the first charge–discharge cycle and after prolonged cycling revealed variable constituents, which indicated the occurrence of several consecutive and parallel processes, including the lithium migration, diffusion, and accumulation. Gnedenkovz and Sinebryukhov investigated through EIS the effect of rutile and/or anatase formation after different chemical/thermal treatment on titanium substrate. Equivalent circuits were proposed to take into consideration the effect of the space charge region as well as the peculiarities of the structure and morphology of the oxide film formed onto titanium surface.^59^ With respect to αTiP/MgSO_4_(MF), its EEC resembled the one found for αTiP.

Table 4 shows the values of the electrical components in each EEC. Although some circuits were built in a similar way, the values of the elements were different. With respect to the resistor element, if compared its values of αTiP with ETiP, ETiP/OHMgSO_4_, and ETiP/EMgSO_4_ (wet via intercalation) it is possible to see that values of modifying sample is between 10 and 10^2^ lower in magnitude. If the comparison is made with αTiP/MgSO_4_(MF) (dry handling mixing) the value showed similar magnitude. When the comparison is made among MgSO_4_ and ETiP, ETiP/OHMgSO_4_ and ETiP/EMgSO_4_, the *R* values were 10^3^–10^4^ times lower in magnitude. If αTiP/MgSO_4_(MF) is considered, the magnitude of *R* value is around 0.6 × 10^2^ lower. The capacitor element (electrostatic storage) was replaced by the Warburg impedance element (mass transport/diffusion of ions or molecules) in ETiP/OHMgSO_4_ and ETiP/EMgSO_4_ owing to magnesium salt. Independent on the route of preparation—wet intercalation or dry handling mixing—all samples decreased the values of constant phase element (Q) (represents a nonideal capacitor). Summarizing, if it is considered that for an EEC the capacitor models electrostatic charge storage contrasting with Warburg element which it is associated with movement/diffusion of ions or molecules, it is reasonable to infer that the wet intercalation was in some extent successful, confirming still that for both ETiP/OHMgSO_4_ and ETiP/EMgSO_4_ magnesium salt was introduced into ETiP lamellae. Still, the results denoted that the higher presence of water in the ethylamine aqueous solution when compared with ethanol had great influence on the ionic conductivity as seen in the plot of current versus voltage.

**Table 4:** Data Description of EEC Components

sample	*R* (kΩ)	*C* (pF)	*Q* (nMho.ŝN)	*W* (fMho.s^1/2^)
αTiP	301	34.4	405	-
MgSO_4_	13.8 × 10^3^	-	70.2 × 10^3^	192
ETiP	29.1	29.8	1.10	-
ETiP/OHMgSO_4_	29	-	1.60	37.7
ETiP/EMgSO_4_	4.4	-	1,78	101
αTiP/MgSO_4_ (MF)	231	26	2.50	-

## Conclusion

This work intended to investigate the intercalation of magnesium sulfate into lamellar titanium phosphate. The effects of wet and dry handling mixing intercalation on the properties were assessed. DRX revealed that ETiP maintained its lamellar spacing even after insertion of MgSO_4_. The latter was dissociated in samples obtained by liquid medium while in dry handling mixing both αTiP and MgSO_4_ retained the integrity of the crystalline arrangement. SEM/EDS indicated that the insertion of MgSO_4_ was more effective in the sample where intercalation used ethylamine aqueous solution. By TGA, it was hypothesized that there is an interaction between amine and sulfate groups meaning better thermal stability endorsed in Raman spectroscopy by the appearance of a new vibration mode at 661 cm^–1^. Spin-lattice and spin-spin relaxation corroborated that molecular mobility of ions magnesium and sulfate was driven by the presence of the ethylamine aqueous solution. EIS evidenced that precursors and dry handling mixing sample are the insulator while the samples intercalated with ethylamine aqueous solution and magnesium salt presented electrical conductivity. The work continues at IMA.

## Data Availability

All data generated or analyzed during this study are included in this published article
